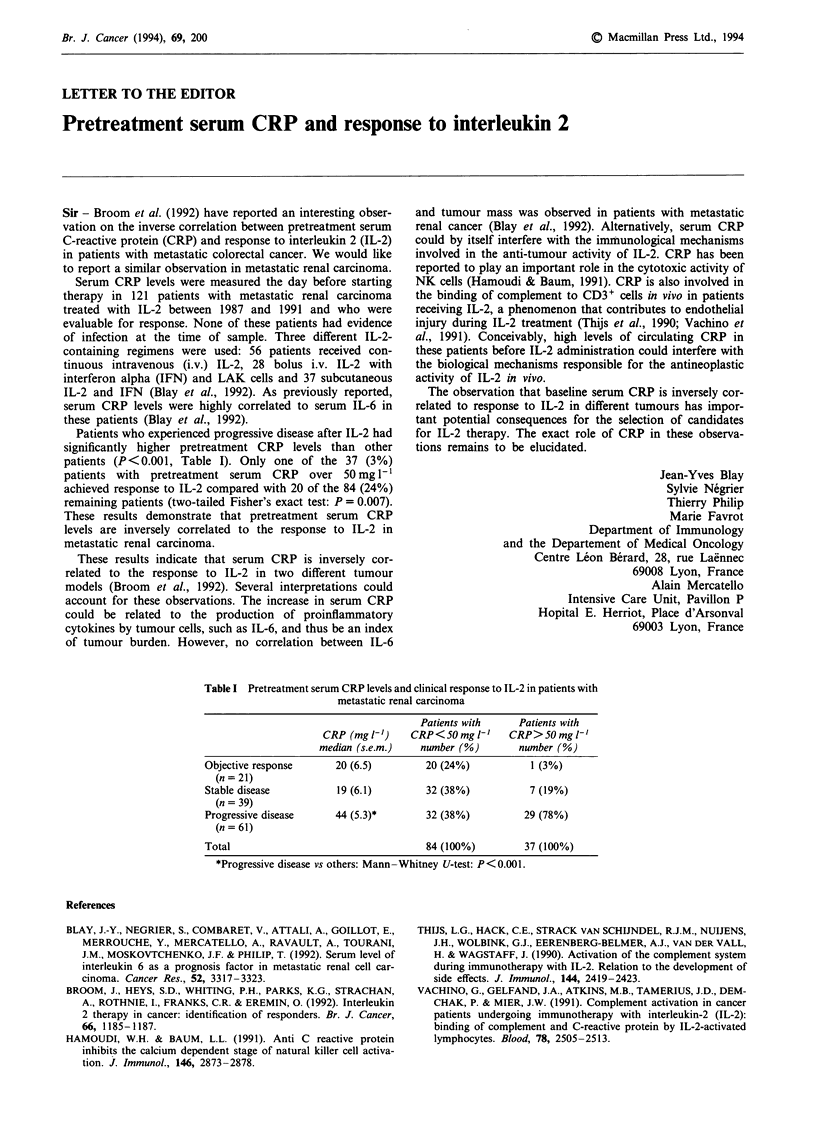# Pretreatment serum CRP and response to interleukin 2.

**DOI:** 10.1038/bjc.1994.35

**Published:** 1994-01

**Authors:** J. Y. Blay, S. Négrier, T. Philip, M. Favrot, A. Mercatello


Br. J. Cancer (1994), **69**, 200 © Macmillan Press Ltd., 1994

LETTER TO THE EDITOR

### Pretreatment serum CRP and response to interleukin 2

**Sir** – Broom *et al.* (1992) have reported an interesting observation on the inverse correlation between pretreatment serum C-reactive protein (CRP) and response to interleukin 2 (IL-2) in patients with metastatic colorectal cancer. We would like to report a similar observation in metastatic renal carcinoma.

Serum CRP levels were measured the day before starting therapy in 121 patients with metastatic renal carcinoma treated with IL-2 between 1987 and 1991 and who were evaluable for response. None of these patients had evidence of infection at the time of sample. Three different IL-2-containing regimens were used: 56 patients received continuous intravenous (i.v.) IL-2, 28 bolus i.v. IL-2 with interferon alpha (IFN) and LAK cells and 37 subcutaneous IL-2 and IFN (Blay *et al.*, 1992). As previously reported, serum CRP levels were highly correlated to serum IL-6 in these patients (Blay *et al.*, 1992).

Patients who experienced progressive disease after IL-2 had significantly higher pretreatment CRP levels than other patients ($P<0.001$, Table I). Only one of the 37 (3%) patients with pretreatment serum CRP over 50 mg $l^{-1}$ achieved response to IL-2 compared with 20 of the 84 (24%) remaining patients (two-tailed Fisher's exact test: $P = 0.007$). These results demonstrate that pretreatment serum CRP levels are inversely correlated to the response to IL-2 in metastatic renal carcinoma.

These results indicate that serum CRP is inversely correlated to the response to IL-2 in two different tumour models (Broom *et al.*, 1992). Several interpretations could account for these observations. The increase in serum CRP could be related to the production of proinflammatory cytokines by tumour cells, such as IL-6, and thus be an index of tumour burden. However, no correlation between IL-6 and tumour mass was observed in patients with metastatic renal cancer (Blay *et al.*, 1992). Alternatively, serum CRP could by itself interfere with the immunological mechanisms involved in the anti-tumour activity of IL-2. CRP has been reported to play an important role in the cytotoxic activity of NK cells (Hamoudi & Baum, 1991). CRP is also involved in the binding of complement to $CD3^+$ cells *in vivo* in patients receiving IL-2, a phenomenon that contributes to endothelial injury during IL-2 treatment (Thijs *et al.*, 1990; Vachino *et al.*, 1991). Conceivably, high levels of circulating CRP in these patients before IL-2 administration could interfere with the biological mechanisms responsible for the antineoplastic activity of IL-2 *in vivo*.

The observation that baseline serum CRP is inversely correlated to response to IL-2 in different tumours has important potential consequences for the selection of candidates for IL-2 therapy. The exact role of CRP in these observations remains to be elucidated.

Jean-Yves Blay
Sylvie Négrier
Thierry Philip
Marie Favrot
Department of Immunology
and the Departement of Medical Oncology
Centre Léon Bérard, 28, rue Laënnec
69008 Lyon, France
Alain Mercatello
Intensive Care Unit, Pavillon P
Hopital E. Herriot, Place d'Arsonval
69003 Lyon, France

**Table I** Pretreatment serum CRP levels and clinical response to IL-2 in patients with metastatic renal carcinoma

| | *CRP (mg $l^{-1}$) median (s.e.m.)* | *Patients with CRP$<$50 mg $l^{-1}$ number (%)* | *Patients with CRP$>$50 mg $l^{-1}$ number (%)* |
|---|---|---|---|
| Objective response ($n = 21$) | 20 (6.5) | 20 (24%) | 1 (3%) |
| Stable disease ($n = 39$) | 19 (6.1) | 32 (38%) | 7 (19%) |
| Progressive disease ($n = 61$) | 44 (5.3)* | 32 (38%) | 29 (78%) |
| Total | | 84 (100%) | 37 (100%) |

*Progressive disease *vs* others: Mann–Whitney *U*-test: $P<0.001$.

### References

BLAY, J.-Y., NEGRIER, S., COMBARET, V., ATTALI, A., GOILLOT, E., MERROUCHE, Y., MERCATELLO, A., RAVAULT, A., TOURANI, J.M., MOSKOVTCHENKO, J.F. & PHILIP, T. (1992). Serum level of interleukin 6 as a prognosis factor in metastatic renal cell carcinoma. *Cancer Res.*, **52**, 3317–3323.

BROOM, J., HEYS, S.D., WHITING, P.H., PARKS, K.G., STRACHAN, A., ROTHNIE, I., FRANKS, C.R. & EREMIN, O. (1992). Interleukin 2 therapy in cancer: identification of responders. *Br. J. Cancer*, **66**, 1185–1187.

HAMOUDI, W.H. & BAUM, L.L. (1991). Anti C reactive protein inhibits the calcium dependent stage of natural killer cell activation. *J. Immunol.*, **146**, 2873–2878.

THIJS, L.G., HACK, C.E., STRACK VAN SCHIJNDEL, R.J.M., NUIJENS, J.H., WOLBINK, G.J., EERENBERG-BELMER, A.J., VAN DER VALL, H. & WAGSTAFF, J. (1990). Activation of the complement system during immunotherapy with IL-2. Relation to the development of side effects. *J. Immunol.*, **144**, 2419–2423.

VACHINO, G., GELFAND, J.A., ATKINS, M.B., TAMERIUS, J.D., DEMCHAK, P. & MIER, J.W. (1991). Complement activation in cancer patients undergoing immunotherapy with interleukin-2 (IL-2): binding of complement and C-reactive protein by IL-2-activated lymphocytes. *Blood*, **78**, 2505–2513.